# Aspect-based classification of vaccine misinformation: a spatiotemporal analysis using Twitter chatter

**DOI:** 10.1186/s12889-023-16067-y

**Published:** 2023-06-21

**Authors:** Heba Ismail, Nada Hussein, Rawan Elabyad, Salma Abdelhalim, Mourad Elhadef

**Affiliations:** 1 College of Engineering, Computer Science and Information Technology Department, Abu Dhabi University, Abu Dhabi, United Arab Emirates; 2 College of Engineering, Electrical, Computer, and Biomedical Engineering Department, Abu Dhabi University, Abu Dhabi, United Arab Emirates

**Keywords:** COVID-19 vaccine, Misinformation, Twitter, Aspect-based analysis, LightGBM, Machine learning

## Abstract

**Background:**

The spread of misinformation of all types threatens people’s safety and interrupts resolutions. COVID-19 vaccination has been a widely discussed topic on social media platforms with numerous misleading and fallacious information. This false information has a critical impact on the safety of society as it prevents many people from taking the vaccine, decelerating the world’s ability to go back to normal. Therefore, it is vital to analyze the content shared on social media platforms, detect misinformation, identify aspects of misinformation, and efficiently represent related statistics to combat the spread of misleading information about the vaccine. This paper aims to support stakeholders in decision-making by providing solid and current insights into the spatiotemporal progression of the common misinformation aspects of the various available vaccines.

**Methods:**

Approximately 3800 tweets were annotated into four expert-verified aspects of vaccine misinformation obtained from reliable medical resources. Next, an Aspect-based Misinformation Analysis Framework was designed using the Light Gradient Boosting Machine (LightGBM) model, which is one of the most advanced, fast, and efficient machine learning models to date. Based on this dataset, spatiotemporal statistical analysis was performed to infer insights into the progression of aspects of vaccine misinformation among the public. Finally, the Pearson correlation coefficient and p-values are calculated for the global misinformation count against the vaccination counts of 43 countries from December 2020 until July 2021.

**Results:**

The optimized classification per class (i.e., per an aspect of misinformation) accuracy was 87.4%, 92.7%, 80.1%, and 82.5% for the “Vaccine Constituent,” “Adverse Effects,” “Agenda,” “Efficacy and Clinical Trials” aspects, respectively. The model achieved an Area Under the ROC Curve (AUC) of 90.3% and 89.6% for validation and testing, respectively, which indicates the reliability of the proposed framework in detecting aspects of vaccine misinformation on Twitter. The correlation analysis shows that 37% of the countries addressed in this study were negatively affected by the spread of misinformation on Twitter resulting in reduced number of administered vaccines during the same timeframe.

**Conclusions:**

Twitter is a rich source of insight on the progression of vaccine misinformation among the public. Machine Learning models, such as LightGBM, are efficient for multi-class classification and proved reliable in classifying vaccine misinformation aspects even with limited samples in social media datasets.

## Background

The last two years have been challenging to humanity as we all fight a global pandemic. With the fast spread of the novel coronavirus and the emergence of new variants world-wide, it was prime for scientists to discover vaccines to prevent and reduce the impact of the virus. In a quest to develop a suitable vaccine, many countries participated in phase 3 clinical trials around November 2020 [1]. Countries like the United States of America (USA), Canada, and the United Kingdom (UK) have registered 93, 22, and 25 clinical trials, respectively [1]. Amongst all the efforts, AstraZeneca produced by the University of Oxford provided a breakthrough with a success rate of 70.4% followed by Pfizer and BioNTech that were able to reduce the severity of 95% of the cases [2]. Statistics show a decline of approximately 84% in COVID-19 reported deaths during the vaccination process in the USA from December 2020 to July 2021 [3]. Similarly, the reported deaths declined by 96% in the UK throughout the same period [3]. As these vaccines performed well, countries like the USA and the UK began mandating the vaccines for public use; however, not a majority of the population supported the intake of a newly discovered and quickly tested vaccine. Simultaneously, new variants of the virus started spreading. Studies show that only 26% of the total hospitalizations of Delta variant in the US are of those who have taken the primary shots and 1% for those who have also taken the booster dose [4]. Similarly, the total hospitalizations of Omicron variant in the US are 33% and 12% for those who have taken the primary shots and those who have taken the booster dose, respectively [4]. However, the effectiveness of the vaccines was still a major concern.

Consequently, throughout and after the development of the various COVID-19 vaccines, the public continuously expressed their concerns, beliefs, and experiences regarding the vaccine jabs on social media platforms. These include the risk of severe events such as clots [5], the effectiveness, especially for children and elderly, as well as the long-term effects of various types of vaccines [6]. Twitter was among the most commonly used platforms for such public discussions, where people freely express and reflect their opinions and viewpoints. With the increasing number of vaccine-related tweets, an increase of shared misinformation was continuously reported. As a result, Twitter removed over 8400 tweets to control the spread of vaccine misinformation content amongst its users [7], while World Health Organization (WHO) released a toolkit to tackle vaccine misconceptions and introduced a hashtag (#VaccinesWork) that would bust myths and delusions regarding the vaccine [8]. Although efforts have been applied by health organizations to reduce the circulation of such messages, it is not adequate to eradicate people’s negative notions towards the vaccine.

Various research efforts attempted to analyze the public opinion and attitude towards the available vaccines. Most of the research work reviewed utilized sentiment analysis to provide insight into the growing anti-vaccine movements on Twitter [9]. For instance, Nezhad et al. [10] utilized sentiment analysis to classify the polarity of Persian COVID-19 vaccine tweets into positive, negative, and neutral. The proposed framework aims to understand the Iranian views and opinions towards local and imported vaccines. Although this proposed framework has proven efficiency, it was limited in geographical coverage as well as in time; the data was collected in a span of 5 months starting from April 2020. Furthermore, Marcec et al. [11] deployed sentiment analysis to provide insight into the public acceptance to three COVID-19 vaccines, namely, AstraZeneca/Oxford, Pfizer/BioNTech and Moderna. This study was focused on providing temporal and causal analytics in the span of 4 months starting from December 2020. This study was limited in time as well as vaccine types. The majority of these research studies were limited either in the timeframe [10, 11], geographical coverage [10, 12, 13] or to certain vaccines [11]. It is critical to take the timeframe, regional scope, and vaccine types into account to fully comprehend the development of vaccine misinformation. The timeframe should be in line with the vaccine launching and administration timeframe. The more comparable the timeframe of the dataset and the vaccine administration, the more insightful the analysis will be. It helps in analyzing timely events and their relevance to the progression of misinformation. Additionally, wider geographical coverage produces more insightful results because different types of COVID-19 vaccines were authorized for use in different countries. Furthermore, since COVID-19 social media hashtags are accessible globally, misinformation that originates in one country can impact other countries. Finally, to fully analyze the progression of misinformation, it is crucial to look at the various vaccine types. We speculate that some vaccines are more frequently and closely related to specific elements of misinformation. Therefore, a larger study's breadth can result in more thorough insights. Therefore, in an attempt to overcome some of these limitations, Yousefinaghani et al. [14] deployed Lexicon-based sentiment analysis to gain insight into the public attitude towards the COVID-19 vaccine on a large scale and over an extended timeframe. The proposed framework tracks the frequent hashtags and discussion topics as well as the progression of themes. Furthermore, the proposed framework compares the opinions from several locations and categorizes the opinions in the tweets into pro-vaccine, anti-vaccine, and hesitant. However, the study does not associate the revealed opinion with specific aspects of misinformation or sources of concern.

Even though some timeframe and geographical coverage limitations have been addressed, the existing frameworks do not provide full in-depth insight into public concerns [15]. In addition, despite the urgency and necessity of addressing the spread of misinformation, very few research works targeted misinformation related to COVID-19 vaccines on social media. Wonodi et al. [16] conducted interviews with representative health and management figures to provide some understanding of the sources of vaccine hesitation, misinformation, and conspiracy theories in Nigeria. However, in these times and under such circumstances, the necessity of automating the analysis process arises. Hayawi et al. [17] proposed the use of multiple Machine Learning (ML) models, including XGBooster, LSTM, and a pre-trained BERT model, to detect COVID-19 vaccine misinformation. Although this framework proved efficient in detecting misinformation and provides some insight, it still lacks in-depth insight into the sources of the spread of misinformation. Sentiment and opinion analysis provide an abstract and broad understanding of the problem and public concerns, which might be enough for tackling some issues; however, this is not the case for vaccine misinformation. In order to provide full insight and perspective to stakeholders, a better understanding of the sources of the concerns and the aspects of the misinformation is needed. To this end, this paper proposes a novel framework for detecting and categorizing aspects of COVID-19 vaccine misinformation on Twitter. The proposed framework provides a comprehensive and solid understanding of the the aspects of the vaccine misinformation among the public. Moreover, it provides a solid understanding of the spatiotemporal progression of the common misinformation aspects related to the various available vaccines. This is achieved through providing analytics factored by the vaccine type, time, and location. This research spans a period of about 7 months, including the introduction and widespread administration of the majority of the common vaccines. Additionally, this research takes into account the analysis of data from Twitter chatter centered on the 10 most popular vaccines.

This work aims to support stakeholders around the globe to combat COVID-19 vaccine misinformation through various representative and comprehensive analytical results. To the authors’ best knowledge, this is the first study analyzing and modeling categories of misinformation through aspect analysis on COVID-19 vaccine Twitter data. In addition, this study publishes the first annotated dataset for aspects of vaccine misinformation. As summarized in Table 1, the reported studies related to COVID-19 vaccine analyze either sentiment or misinformation only, but not the sources and aspects of misinformation. Furthermore, only a few studies published the related datasets.

**Table 1:** Review of existing research works related to COVID-19 vaccine

Research Work	Method	Misinformation/ Sentiment/ Aspects of Misinformation	Published Dataset
Alam et al. [9]	Deep Learning-Based Sentiment Analysis	Sentiment	No
Nezhad et al. [10]	Deep Learning-Based Sentiment Analysis	Sentiment	Not mentioned – collected a Persian dataset
Marcec et al. [11]	Lexicon-Based Sentiment Analysis	Sentiment	Not mentioned
Bustos et al. [12]	Statistical Sentiment Analysis	Sentiment	Not mentioned
Alabrah et al. [13]	Machine Learning-Based Sentiment Analysis	Sentiment	No
Yousefinaghani et al. [14]	VADER; Lexicon-Based Sentiment Analysis	Sentiment	No
Wonodi et al. [16]	Focus group discussions and informant interviews	Misinformation	No – Available upon request
Hayawi et al. [17]	ML-Based Sentiment Analysis	Misinformation	Yes

## Methods

**Fig. 1:**
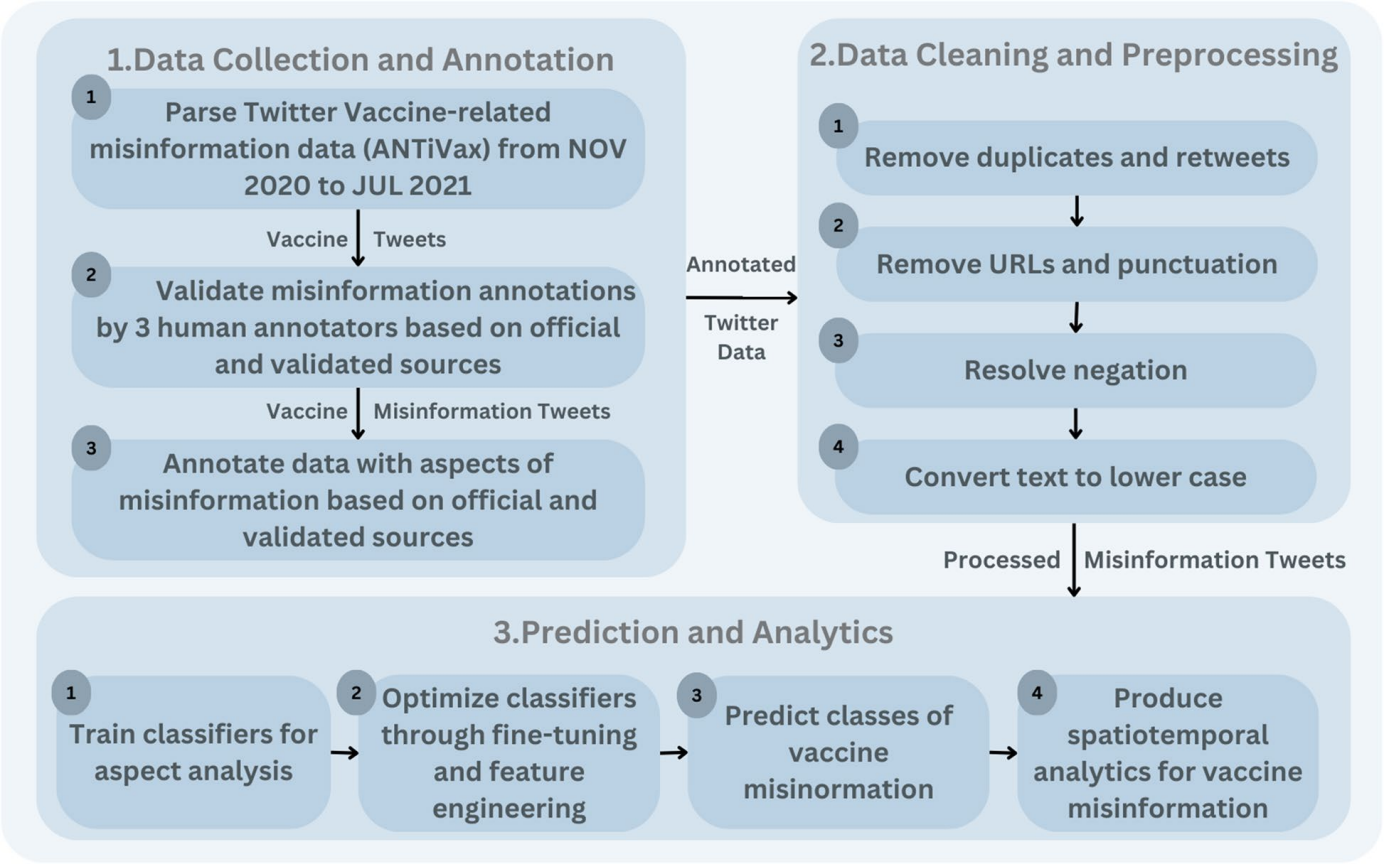
CovVax misinformation aspects analytics framework

This section describes the methodology of the proposed vaccine misinformation aspect analysis framework. As illustrated in Fig. 1, the pipeline starts with data collection and annotation, followed by cleaning and preprocessing, finally, the prediction and the analytics, including the training, validation, and evaluation of the Machine Learning models used.

### Dataset

To annotate a training dataset for COVID-19 vaccine misinformation aspects, we utilized the ANTi-Vax English dataset containing COVID-19 vaccine-related tweet IDs collected and organized between December 20, 2020 to July 21, 2021 [17, 18]. This dataset was collected by filtering Twitter data using a predefined set of keywords, including: ‘vaccine’, as well as specific COVID-19 vaccine names such as ‘Sinopharm’, ‘Moderna’, etc. This dataset was introduced as a vaccine misinformation dataset, which serves the purpose of the proposed framework. Moreover, the timeline of data collection is sufficient to provide insight into the progression and evolution of misinformation aspects. The dataset is collected over a span of approximately 7 months, starting with the approval of the first vaccine in December 2020 and covering the approvals and distribution of the common vaccines, namely, Pfizer, AstraZeneca, Sinopharm, Sputnik, Moderna, Johnson&Johnson, Covaxin, Covishield, SARS-CoV-2, Sinovac.

Since only the tweets IDs and labels were published, the IDs were hydrated to extract the full tweet’s content for every corresponding tweet ID in a comma-separated values file (CSV). The resulting CSV file includes the text of the tweets as well as related metadata, e.g., location and timestamp, which are necessary for spatiotemporal analytics. After the hydration process, approximately 12 k tweets are parsed, 4 k of which were labeled as by the authors as misinformation.

### Data annotation

The annotation task was conducted over two stages. First, misinformation labels annotated by the authors of the published misinformation dataset were manually validated. Second, aspects of misinformation were categorized, labeled, and verified relevant to verified medical sources. Although the ANTi-Vax Twitter dataset [17, 18], was annotated for misinformation detection, multiple misclassifications were detected during the manual validation of the dataset. Therefore, the data was re-annotated manually for misinformation detection.

Table 2 shows the rules used for both annotation stages. These rules were obtained from reliable medical resources, including the Centers for Disease Control and Prevention (CDC) [19, 20]. The guidelines were developed based on a Toolkit from UNICEF [21], similar annotation guides [22, 23], as well as policies and guides used by Twitter [24]. The aspects’ guidelines were refined throughout the process of annotation to achieve comprehensive coverage of all discussed topics related to vaccine misinformation concerning the public on Twitter.

**Table 2:** Annotation rules and guidelines used to categorize aspects of misinformation

Annotation Rules and Guidelines	
**Aspect #1** **Vaccine Constituent**	**Aspect #2** **Adverse Effects**
• The ingredients of the vaccine are similar to those in many foods – fats, sugars, and salts • The vaccine includes preservatives, tissues (like aborted fetal cells, antibiotics, food proteins, medicines, latex, or metals • The mRNA vaccine is not considered a vaccine • The mRNA vaccine is gene therapy • This vaccine is gene therapy, not an actual vaccine • COVID-19 vaccines authorized for use shed or release their components • The vaccine contains magnetic materials like metals that make the vaccinated area electromagnetic • The vaccine contains HIV, Ebola, or Botulism • The vaccine is full of toxic ingredients	• The vaccine can lead to the development of the coronavirus infection • The vaccine can cause variants of the virus • The vaccine causes adverse health conditions such as heart attacks • The vaccine causes adverse allergic reactions • The vaccine’s adverse can even lead to death • The vaccine can lead to fainting or passing out • All events reported to the Vaccine Adverse Event Reporting System (VAERS) are caused by vaccination • The reproductive system of both males and females may be negatively impacted • The vaccine can lead to a delay in menstrual cycles, painful cramps, and hormonal imbalance • The vaccine can cause fertility problems in both genders People trying to get pregnant should not take the vaccine • Pregnant females should not take the vaccine • Breastfeeding mothers should not take the vaccine
**Aspect #3** **Agenda**	**Aspect #4** **Efficacy and Clinical Trials**
• More than the virus, the vaccine is deadly and can kill people, accounting for depopulation • The vaccine fits in a plan to kill certain ethnicities or groups of people • The vaccine is a bioweapon • The vaccine can cause DNA alteration or is part of gene therapy • The vaccine has a microchip embedded within it that may be injected into the body • The microchip is meant to track patient data • The microchip is meant to control the population • The vaccine affects fertility in a plan for depopulation • The vaccine is only meant for profit	• The vaccine clinical trials are not complete • The clinical trials compromise science • The vaccines are not adequately tested • There are no peer-reviews or data to support the efficacy of the vaccine • Illegal and unproven vaccine • The vaccine is fast-tracked, experimental, and liability-free • The vaccine was never tested on humans • The vaccine is not effective at all • The vaccine cannot prevent sickness or reduce symptoms • The vaccine cannot stop or reduce the spread • The vaccine is not effective with new variants at all • Human immunity can prevent COVID-19 better than the vaccine

Throughout the first stage, the tweets were read manually and annotated as misinformation if they explicitly or implicitly contain any of the myths or misinformation in the rules and guidelines. For instance, Table 3 shows samples of tweets that were annotated as misinformation or otherwise.

**Table 3:** First annotation stage: sample tweets annotated as misinformation vs not misinformation

Misinformation	Not Misinformation
“The vaccine makes you infertile lol…depopulation with no deaths? Well not counting COVID deaths but you know”	“If your pillow looks like this, don’t worry about what’s in the vaccine”
“Don’t you dare take an mRNA experimental #vaccine and you are pregnant!!! There is no data that says it’s safe and effective for pregnant women or their unborn children.”	“I’m so excited!! I just signed up and received my ticket for the COVID vaccine! Together we will beat this devil ”
“Well, it happened. My daughter used my grandchildren to try to guilt me in to taking the vaccine for the CCP bioweapon. Satan is disguised in so many forms. Be on your guard…even when it feels like your heart is being ripped out. #StandStrong ”	“Moderna mRNA #Covidvaccine published results now released. Really impressive data in terms of effectiveness (94%). Very excited to see this in the arms of those in remote, LTC communities, others soon!

After the first stage of annotation, only tweets including misinformation were annotated again in the second stage to identify the misinformation aspect. During this stage, the aspects were assigned based on the content of the tweets as well as the text of the hashtags used. Both explicit and implicit aspects were considered in this stage too. The dominant aspect was considered for multi-aspect tweets. Table 4 shows samples related to the four aspects of misinformation.

**Table 4:** Second annotation stage: sample tweets annotated with aspects of misinformation

Aspect	Tweet Samples
Aspect #1 Vaccine Constituent	“Vaccine ingredient: Monosodium Glutamate [MSG]. A toxic chemical that is linked to birth defects, developmental delays and infertility. Banned in Europe.”
Aspect #2 Adverse Effects	“The "V"s spike protein is biologically active and causes blood clots, leading to strokes, heart attacks, pulmonary embolism and infertility effects. Pfizer’s own documents reveal this phenomenon to be well known by vaccine developers.”
Aspect #3 Agenda	“Agenda 21 is underway. It means global genocide via “vaccine” (80% depopulation plan), global communism and one religion. Virus, lockdowns & “vaccine” are pre-planned weapons of this demonic agenda. Ties to spy gate cover-up (AKA crossfire hurricane). Thread https://t.co/0heTaDzoIu”
Aspect #4 Efficacy and Clinical Trials	“Can someone please explain the logic behind healthy individuals getting the #COVID19 vaccine when it poses a nearly 0% chance of death? Plus, you can still transmit it post-vaccination. I'll pass on the rushed vaccine.”

**Fig. 2:**
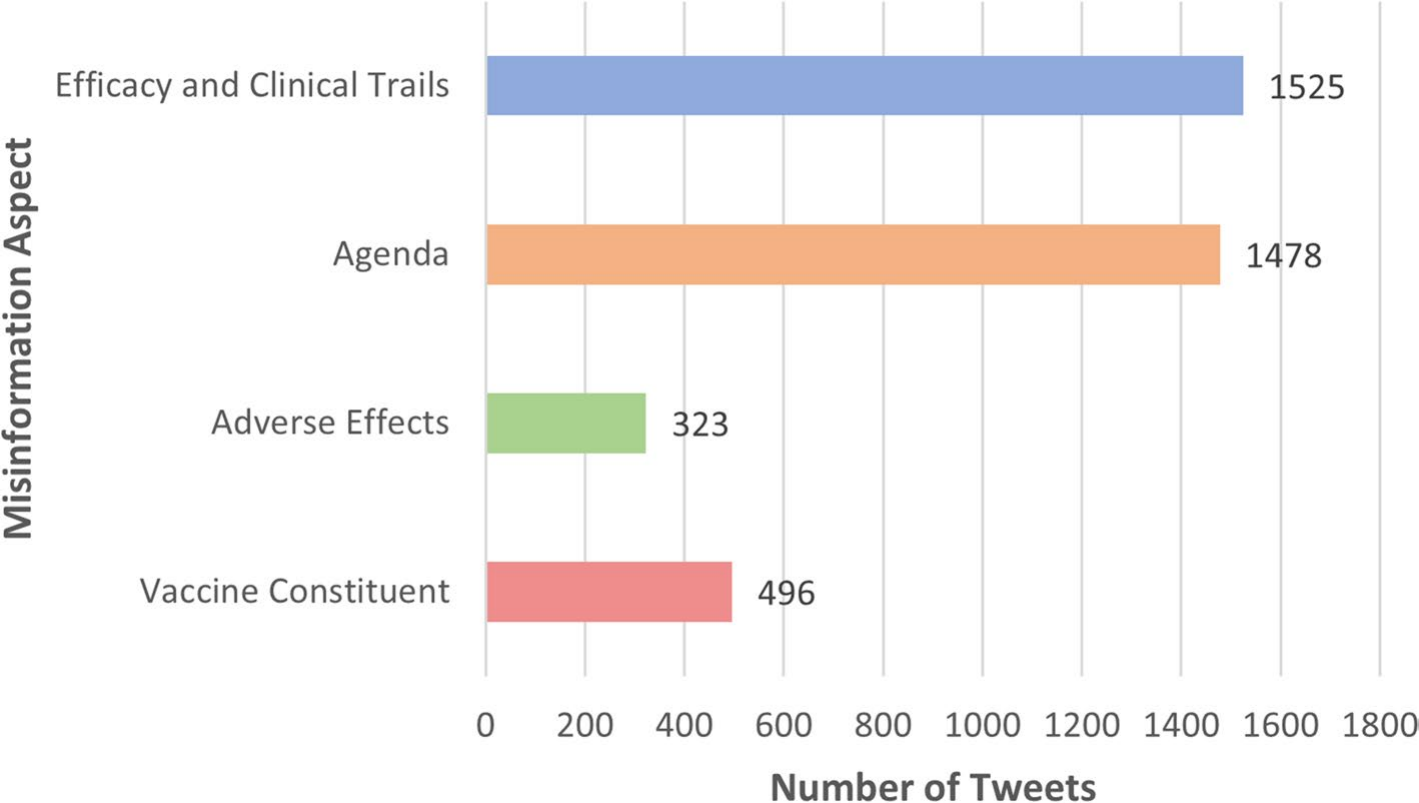
Vaccine misinformation aspects’ distribution

Figure 2 demonstrates the misinformation aspects distribution. It can be seen that “Agenda” as well as “Efficacy and Clinical Trials” were the dominant aspects representing public concerns about the vaccine, making up for almost 80% of the dataset.

### Data preprocessing

The annotated dataset was passed through the preprocessing and cleaning pipeline prior to training the models. First, the pipeline starts by dropping the duplicated tweets in the dataset produced by retweets. Second, the external URLs and links, and punctuation were removed. Third, the negation was resolved. Resolving negation is a common step in the pre-processing pipeline in Natural Language Processing. Negation words such as “no”, “not”, “never”, … etc., can significantly alter the meaning and semantic orientation of a sentence. Therefore, in this step, the framework detects negated words and modifies the sentiment of the following part of the sentence accordingly. However, a predefined set of negation-related stopwords were kept, e.g., don’t, no, not, etc., since negations are of extreme importance for prediction and analytics in the context of misinformation. For instance, it is crucial to detect negations of vaccine efficacy, e.g., “This vaccine is not a true vaccine. It is a trial”. Finally, the text was converted to lowercase.

## Results

### Prediction of aspects of vaccine misinformation

Multiple experimental trials were conducted to achieve the highest possible performance for the aspect prediction model. Out of those models, the optimized LightGBM model with 50 features outperformed the experimental models. This model was trained on the stratified annotated vaccine misinformation dataset with a split of 85% for training and 15% equally split between validation and testing. Table 5 shows the dataset distribution.

**Table 5:** Dataset distribution

Dataset	Number of Tweets
Training	3250
Validation	286
Testing	286

The LightGBM aspect classification model was able to achieve a validation ensemble accuracy of 80.1% and a testing accuracy of 71.3%. Accuracy is the performance evaluation metric used throughout the evaluation of the experimental results. For each of the vaccine misinformation aspects, for instance, “Vaccine Constituent,” the classification results could be any of the following:

- True Positive (TP): Predicted aspect is “Vaccine Constituent” and the ground truth is “Vaccine Constituent.”
- True Negative (TN): Predicted aspect is any non-“Vaccine Constituent” aspect, and the ground truth is any non-“Vaccine Constituent” aspect
- False Positive (FP): Predicted aspect is “Vaccine Constituent” while the ground truth is any non-“Vaccine Constituent” aspect
- False Negative (FN): Predicted emotion is any non-“Vaccine Constituent” aspect, while the ground truth is “Vaccine Constituent.”

Accuracy is the percentage of correctly labeled tweets.

$$Accuracy= \frac{TP+TN}{TP+FP+FN+TN}$$

Table 6 shows the obtained per-class (i.e., per an aspect accuracy).

**Table 6:** Vaccine misinformation aspect experimental results per class

Aspect	Accuracy
**Vaccine Constituent**	87.4%
**Adverse Effects**	92.7%
**Agenda**	80.1%
**Efficacy and Clinical Trials**	82.5%

**Fig. 3:**
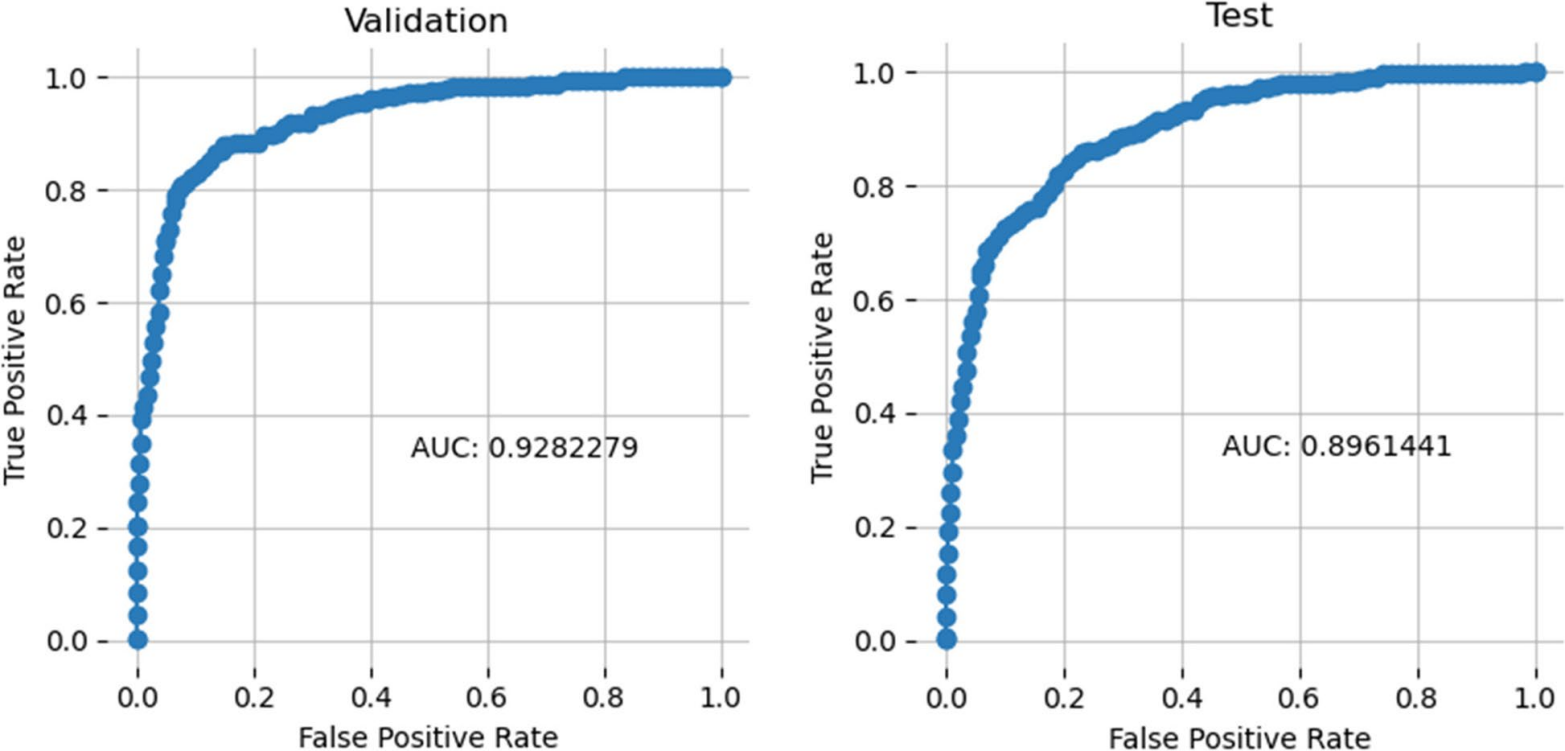
Receiver Operating Characteristic (ROC) Curve

Figure 3 shows the Receiver Operating Characteristic Curve (ROC) of the validation and testing, respectively. The obtained testing Area Under the ROC Curve (AUC) is 90.3% and 89.6% for validation and testing, respectively. These results indicate acceptable performance, considering the skewness of the data.

### Spatiotemporal analytics

**Fig. 4:**
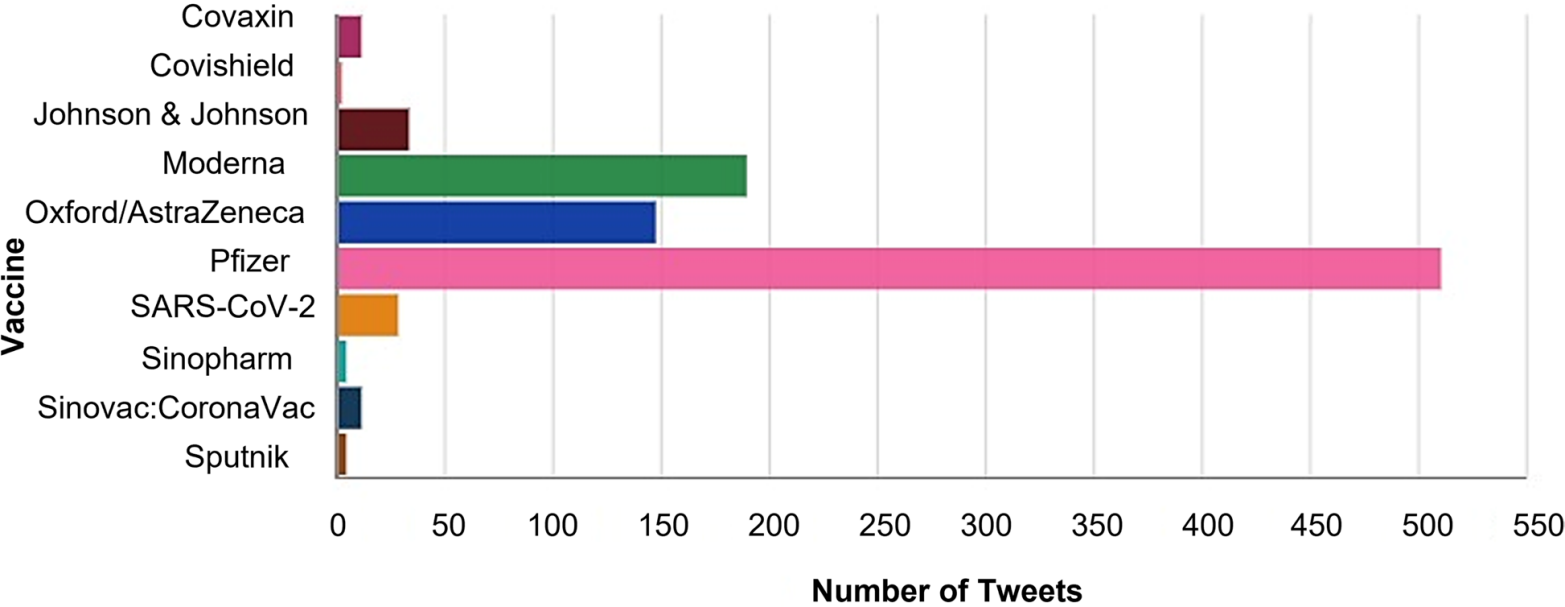
Frequency of misinformation tweets per vaccine type

In order to provide an in-depth insight of the progression of vaccine misinformation aspects, multiple analytics were produced. For instance, Fig. 4 illustrates the number of misinformation tweets associated with each type of vaccine. It can be seen that Pfizer, Moderna, and AstraZeneca had the majority of the public misinformation. Pfizer, specifically, was by far the most discussed in the misinformation tweets relevant to all aspects, making up for approximately 53% of the total tweets in the dataset.

**Fig. 5:**
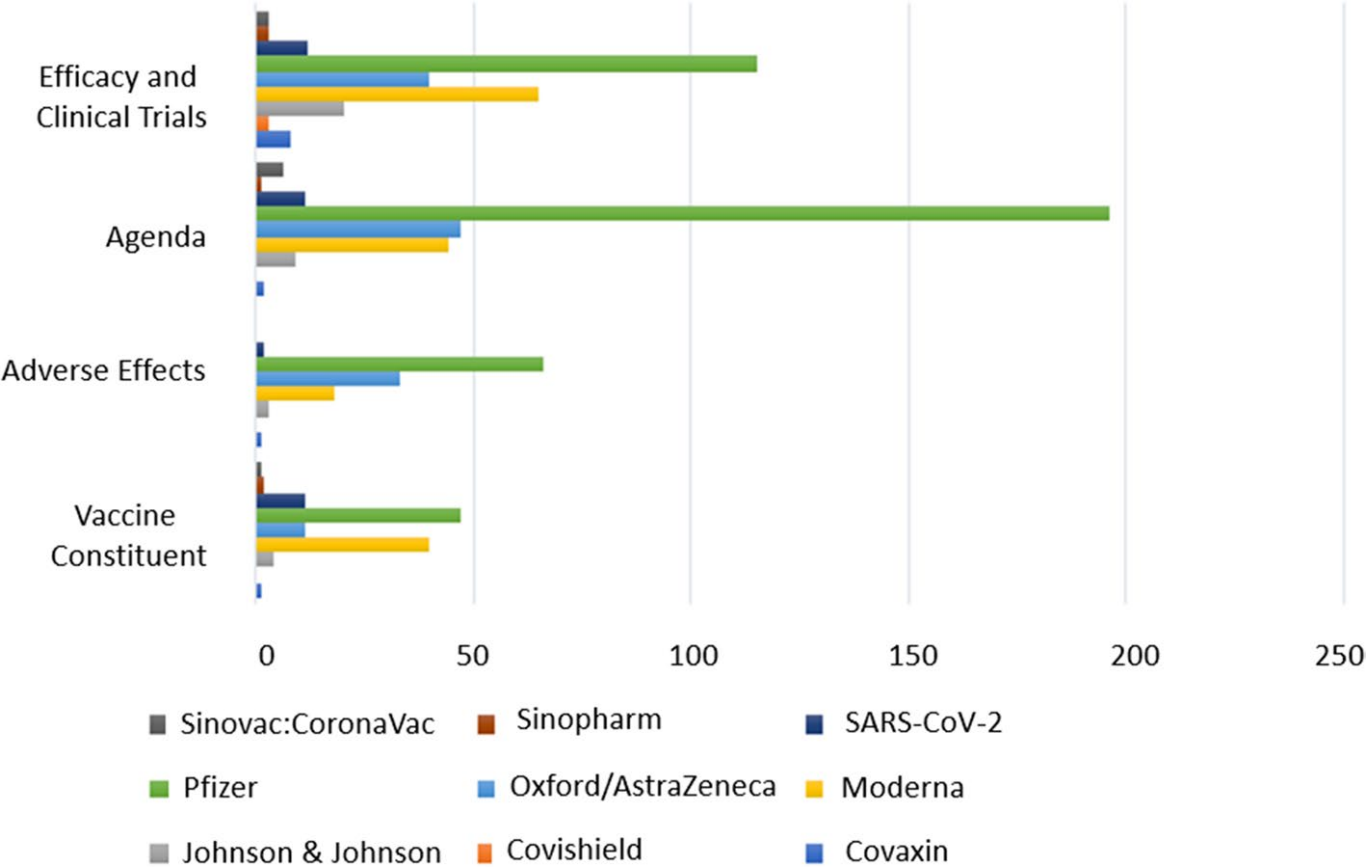
Frequency of misinformation tweets per vaccine and per aspect

Furthermore, Fig. 5 illustrates the number of misinformation tweets factored by aspect for each vaccine type. It can be seen that Pfizer, i.e., represented by the green bar, generated most of the vaccine misinformation discourse relevant to all the aspects. Moreover, Moderna and AstraZeneca, i.e., represented by yellow and light blue bars respectively, were the second most discussed vaccines in the misinformation tweets relevant to all the defined misinformation aspects. However, it is observed that Moderna was specifically associated with the aspects of “Vaccine Constituents” and “Efficacy and Clinical Trials” more frequently than AstraZeneca since it is an mRNA-based vaccine with questionable efficacy among the public. Meanwhile, AstraZeneca was associated more frequently than Moderna with the aspect of “Adverse Affects” given that many cases reported blood clots and heart problems after taking the vaccine. Furthermore, it can be seen that the aspect of “Agenda Discussions” associated with Pfizer was almost three times as frequent as AstraZeneca and exceeded the total frequency of tweets reporting the same aspect of misinformation relevant to all other vaccine types. The fact that Pfizer is an mRNA-based vaccine triggered much discourse among the public, which was reported in their tweets.

**Fig. 6:**
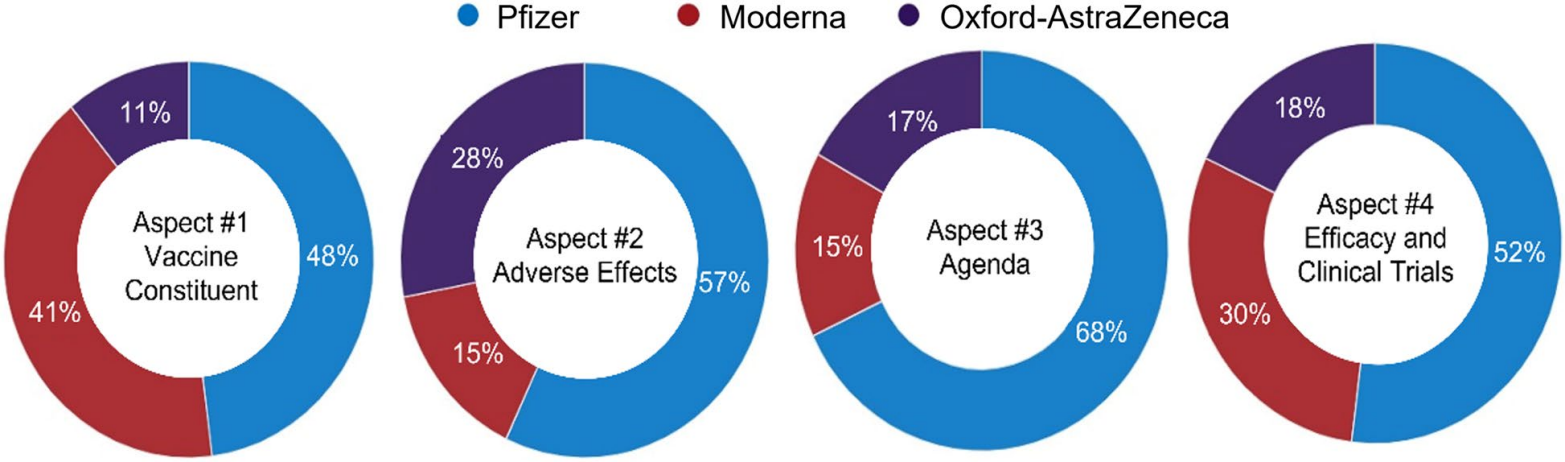
Misinformation aspects associated with the three most common vaccines

Figure 6 shows the percentage of misinformation aspects associated with the three most discussed vaccine types on Twitter: Pfizer, Moderna, and AstraZeneca.

**Fig. 7:**
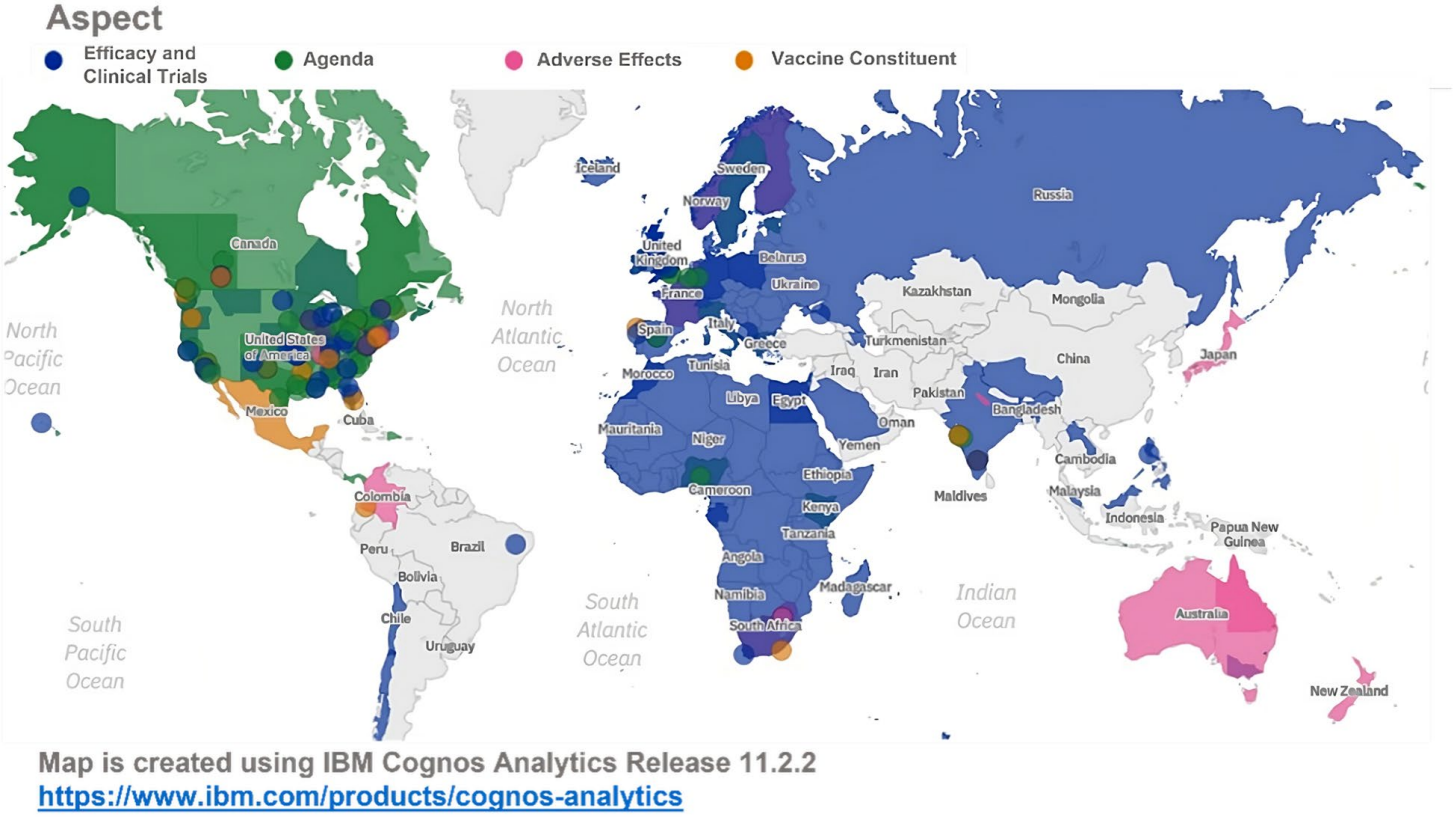
Aspects of vaccine misinformation spatial analysis

To understand the spatiotemporal progression of the vaccine misinformation, the tweets' geo-location, and timestamp metadata of Twitter chatter were used to develop spatial and temporal analytics. To illustrate the geographical spread of the most dominant vaccine misinformation aspects, Fig. 7 shows the spatial distribution obtained from the Twitter data. Figure 7 provides a visual representation of the prevalence of the four vaccine misinformation aspects considered in this research. Blue refers to ‘Efficacy and Clinical Trials’, green refers to ‘Agenda’, pink refers to ‘Adverse Effects, and orange refers to ‘Vaccine Constituent’. The map shows spatial dominance at two levels: coverage and intensity. Regions are colored with a specific color to indicate the dominance of a certain aspect of misinformation in that region. The intensity of the color indicates the frequency or the prevalence of particular aspects of misinformation in specific regions. Furthermore, overlapping colors indicate several aspects of misinformation prevalent in that particular region. Finally, circles, indicate more specific areas such as cities that were mentioned in the Twitter chatter data.

By mapping the spatial distribution of the aspects of misinformation, policymakers can identify regions that require s specific interventions aimed at correcting misguided beliefs with evidence-based messaging.

As illustrated in Fig. 7, the vaccine “Efficacy and Clinical Trials” was the most dominant aspect in European countries as well as Middle Eastern and African countries. With 33 total registered trials in Russia, 18 in South Africa, and less than 10 trials in the rest of Africa [1], Twitter users expressed their growing concern on the insufficient testing of the experimental jabs. It was also observed that “Efficacy and Clinical Trials” as well as “Agenda” misinformation aspects were dominant in South Africa, where some experimental trials were conducted before approving the vaccine. Meanwhile, Twitter users were more concerned about the vaccine being part of an agenda, whether it is depopulation or market profit, in the USA and Canada. Throughout the study duration, most of the tweets concerned about the adverse effects were in Australia, Colombia, and Japan. Several adverse events were reported worldwide and in Australia [25], including testing positive for HIV as many claimed.

**Fig. 8:**
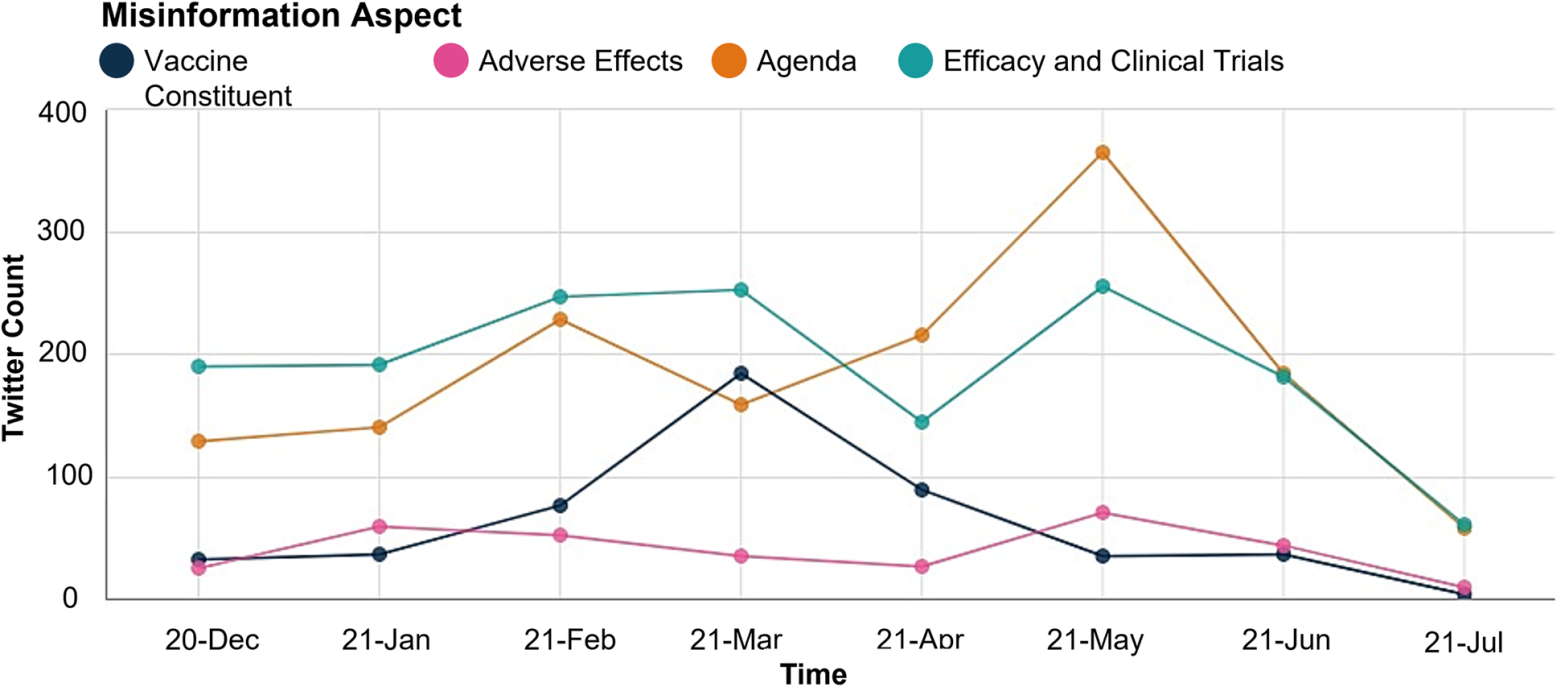
Aspects of vaccine misinformation temporal analysis

Figure 8 shows the temporal progression of misinformation aspects over the dataset timeline, starting from the approval of the first vaccine in December 2020 until July 2021.

As shown in Fig. 8, the “Efficacy and Clinical Trials” was the dominant aspect during the first three months, starting from the first official approval of the vaccine. Post the official approval, and as published by the American Journal of Managed Care (AJMC) [25], many countries, including the UK, began distributing the vaccines. In addition, vaccination acceleration plans were adopted in the USA. In early February and March, multiple efficacy-related reports were published. Simultaneously, multiple variants started to spread, raising the concern about the efficacy of the vaccines.

An increasing public controversy arose in March, associating blood clots with AstraZeneca after several reported cases. In response to those cases, in South Africa and many European countries, the vaccine was temporarily suspended to further investigate the association of the vaccine with blood clots. Moreover, AstraZeneca issued a statement refusing the causality link between its vaccine and blood clots and experts stressed that there is no causal link [25]. This can be seen in the temporal progression of the curve associated with the aspect “Efficacy and Clinical Trials”, represented in green color in Fig. 8. The curve grows between February and March, then, gradually declines toward April upon the release of experimental statistics negating the relationship between the vaccine and the reported cases. Toward the end of March, Pfizer and Moderna released positive efficacy data and survey results reporting a drop in vaccine hesitancy [26]; hence, the decline of the “Efficacy and Clinical Trials” aspect curve.

Furthermore, the “Agenda” aspect, represented by the yellow color in Fig. 8, starts to peak in April. This can be explained by the latest events starting from late April when fake Pfizer vaccines were reported in Mexico and Poland [27] as well as the distribution of vaccines, especially AstraZeneca, outside the USA.

The “Efficacy and Clinical Trials” aspect peaks again during May 2021. May witnessed the preparation for authorization and approval of Pfizer vaccine in adolescents [25]. Moreover, multiple vaccines, including doses of Johnson & Johnson, Pfizer, Moderna, and AstraZeneca—that were not approved yet in the USA- were shipped out from the USA [25]. In addition, several cases of adverse effects, including heart problems, were reported towards the end of May. This is reflected in the analysis, as the “Efficacy and Clinical Trials” as well as “Adverse Effects” aspects peak. Furthermore, it is observed that “Agenda” remains the most dominant aspect and remarkably peaks in May, exceeding the peaks of all aspects throughout the entire duration. The peak of the three previous aspects was aligned due to the relatedness of the topics involved in discussing these aspects. This indicates that the spread of misinformation leads to the public being concerned about having intentionally rushed and untested vaccines that have adverse and dangerous effects that fit into depopulation and profit agendas. Finally, in relation to the timeline, in June 2021, employers in the USA were permitted by the Equal Employment Opportunity Commission to mandate vaccination among their employees [25].

**Fig. 9:**
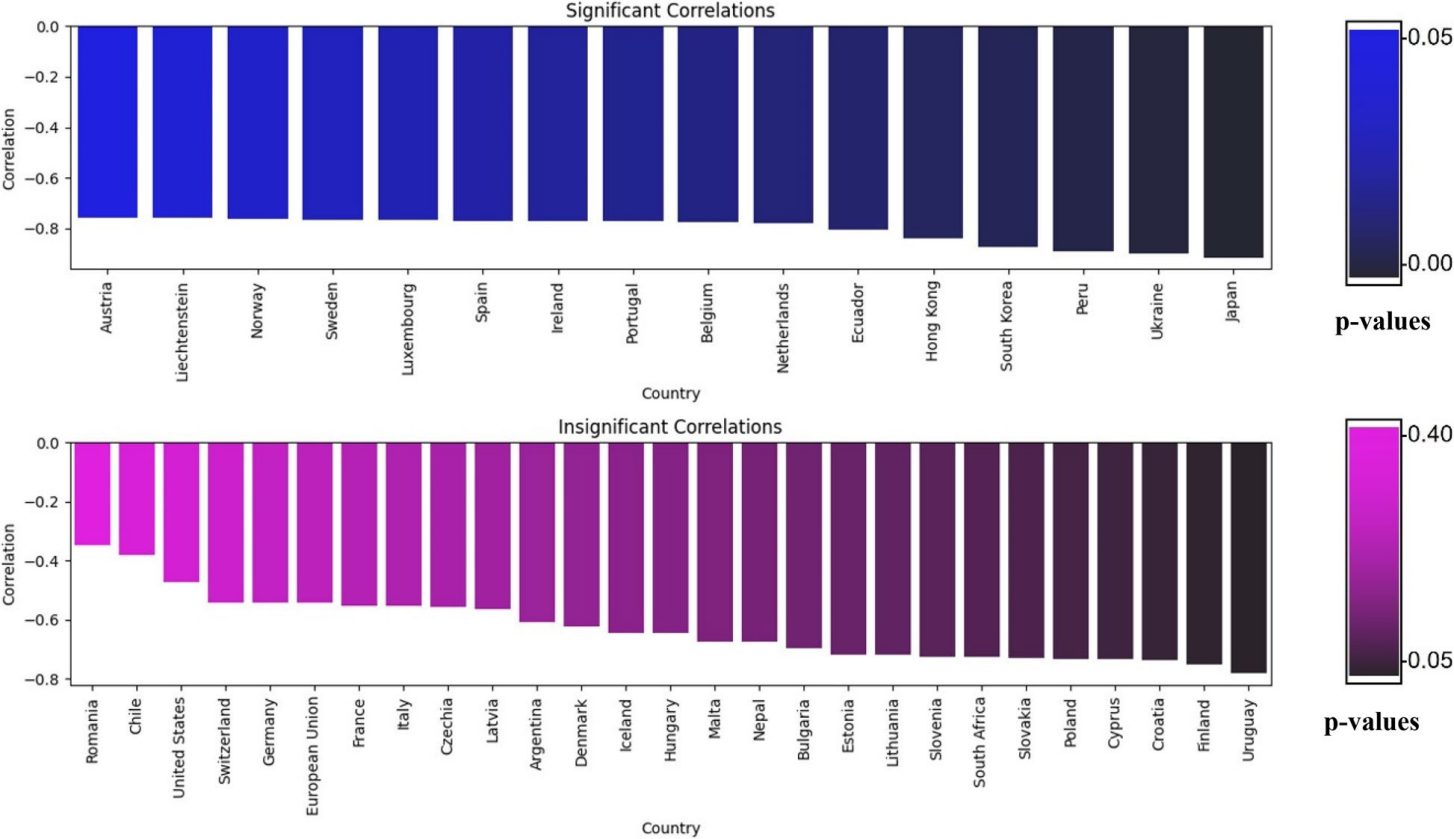
Pearson correlation coefficient of misinformation tweet counts against vaccination counts between December 2020 and July 2021

To evaluate the correlation between the misinformation tweets’ count and the number of vaccinations administered world-wide during the same timeframe, Standard Pearson correlation is calculated. This work hypothesizes that these two variables are negatively correlated at significance level of 0.05. The Pearson correlation coefficient and p-values are calculated for the global misinformation count against the vaccination counts of 43 countries from December 2020 until July 2021. The correlation values varied, but all values indicated negative correlations with values ranging from -0.349 to -0.915. The highest numerical value indicates stronger negative correlation between the study variables, indicating that the increase of misinformation tweet leads to the decrease of vaccinations. To assess the significance of the correlation, a p-value threshold of 0.05 is used. Values equal to or less than 0.05 are considered significant, indicating a strong significant correlation. On the other hand, p-values greater than 0.05 are considered insignificant. Figure 9 plots the correlation of the misinformation count against the vaccination count for 43 countries, and divides them into significant, and insignificant correlations. The gradient shades of the bar charts are proportional to the p-values, where darker values indicate lower p-values, hence, more significant correlation. The graph shows that 37% of the countries were negatively affected by the spread of misinformation. Since misinformation tweets’ spread on social media is globally accessible, we deem it imperative to consider their significance beyond local contexts.

## Discussion

### Main findings

Machine Learning classification models prove reliable in detecting aspects of misinformation from the public chatter on Twitter with high accuracy results, as summarized in Table 6. The spatiotemporal analysis further validates the reliability of the proposed misinformation aspect classification framework. Spatiotemporal analytics are aligned with real-life temporal vaccine-related events. The progression of aspects of the vaccine misinformation on Twitter was directly associated with the reported vaccine-related news in different countries. Certain vaccines were more associated with specific aspects of vaccine misinformation among the public. For instance, throughout the span of this study, Pfizer was associated with the majority of misinformation tweets. These findings prove that decision-makers and policy officials can benefit from the analysis of the progression of vaccine misinformation on social media to promptly understand public concerns and design intervention plans to combat the spread of such misinformation.

### Interpretation

The findings of this study show a clear association and correlation between the progression of vaccine-related misinformation and public vaccine hesitancy. According to global and regional vaccination data, several countries show slower vaccination rates during periods of increased spread of misinformation. On the other hand, the trend lines are steeper, indicating higher vaccination rates, during periods of the decreased spread of misinformation [28]. The public’s opinions reformulate and fluctuate relevant to several life events. Social media is a public venue for the exchange of public opinions. The majority of people do not solicit their information from reliable medical sources and rely on social media as a source of information that influences their actions. Consequently, the spread of misinformation around critical medical resolutions can drastically impact people’s safety and nations’ public health. This results of this work prove the hypothesis that there is significant negative correlation between misinformation spread and vaccination rates for 37% of the countries in the study. Hence, providing instant and accurate insight into the aspects of misinformation on social media can significantly support policymakers in understanding public concerns, hence, taking actions to combat misinformation and raise public awareness.

### Limitations

While this study was comprehensive in terms of spatiotemporal analysis and validation with real-life events, it can be extended to cover a larger timeframe. Moreover, although this study considers vaccine-related tweets worldwide, it was only focused on English tweets which were the most prominent on Twitter. Hence, this study can be further extended to analyze multilingual datasets; tweets in native languages are valuable and would provide fine-grained insights. Furthermore, given the incomparable popularity of Twitter across different regions and populations, the data collected, and the corresponding interpretation may be more representative of regions where Twitter is the most commonly used social media platform. However, this study may not fully reflect the progression of vaccine misinformation for regions where Twitter is not the most popular social media platform. Thus, future work may consider supplementing Twitter chatter data with data from other social media platforms that are more popular in specific regions.

## Conclusion

COVID-19 vaccine hesitancy is a primary worldwide concern since it significantly affects public health. Misinformation contributes significantly to vaccine hesitancy among the public. Social media platforms have witnessed an increasing number of shared misinformation, especially Twitter. That is because people tend to freely and informally express their opinions and share their thoughts on Twitter. Although several global associations and organizations attempted to fight the spread of vaccine misinformation, the efforts were limited. Moreover, the reviewed literature was limited to sentiment analysis of COVID-19 vaccine tweets, with only a few studies focused on misinformation in general but not the specific aspects of misinformation. To this end, this paper is the first to propose a novel Misinformation Aspect Analysis framework that detects and classifies COVID-19 vaccine misinformation into medically verified aspects. The manually annotated dataset of vaccine misinformation used in this paper is publicly available on GitHub. Moreover, this framework produces a variety of spatiotemporal analytics that aim to support several stakeholders in assessing the situation and making positive intervention plans accordingly. These analytics provide timely and in-depth insights into the spatial and temporal progression of vaccine misinformation and sources of concerns.

This framework deployed a LightGBM model for classifying misinformation aspects and achieved per-class accuracies of 87.4%, 92.7%, 80.1%, and 82.5% for the “Vaccine Constituent,” “Adverse Effects,” “Agenda,” “Efficacy and Clinical Trials” aspects, respectively. Furthermore, the model achieved an Area Under the ROC Curve (AUC) of 90.3% and 89.6% for validation and testing, respectively. In addition, the findings and insights derived from the spatiotemporal analytics are consistent with the timeline of events related to the COVID-19 vaccine world-wide, proving the reliability of the proposed model.

## Data Availability

The annotated COVID-19 vaccine misinformation aspects dataset is available for researchers. Only the tweet IDs and labels of misinformation aspects are available, complying with Twitter’s data privacy and terms of service. The dataset is accessible on GitHub through this link: https://github.com/VaccineABSA/CoVax-Aspects/blob/main/README.md.

## Abreviations

ML: Machine Learning
BERT: Bidirectional Encoder Representations from Transformers
LSTM: Long Short-Term Memory
LightGBM: Light Gradient Boosting Machine
TP: True Positive
TN: True Negative
FP: False Positive
FN: False Negative
ROC: Receiver Operating Characteristic Curve
AUC: Area Under the ROC Curve
CSV: Comma-Separated Values
